# Siloxane Containing Polyether Groups—Synthesis and Use as an Anti-Biocorrosion Coating

**DOI:** 10.3390/ijms25126801

**Published:** 2024-06-20

**Authors:** Joanna Karasiewicz, Rafał M. Olszyński, Paulina Nowicka-Krawczyk, Joanna Krawczyk, Łukasz Majchrzycki

**Affiliations:** 1 Department of Chemistry and Technology of Silicon Compounds, Faculty of Chemistry, Adam Mickiewicz University in Poznań, Uniwersytetu Poznańskiego 8, 61-614 Poznan, Poland; 2 Department of Algology and Mycology, Faculty of Biology and Environmental Protection, University of Lodz, Banacha 12/16 Street, 90-237 Lodz, Poland; rafal.olszynski@biol.uni.lodz.pl (R.M.O.); paulina.nowicka@biol.uni.lodz.pl (P.N.-K.); 3 Department of Interfacial Phenomena, Faculty of Chemistry, Maria Curie-Skłodowska University, Maria Curie-Skłodowska Sq. 3, 20-031 Lublin, Poland; joanna.krawczyk@mail.umcs.pl; 4 Institute of Physics, Faculty of Materials Engineering and Technical Physics, Poznan University of Technology, Piotrowo 3, 60-965 Poznan, Poland; lukasz.majchrzycki@put.poznan.pl

**Keywords:** organofunctional siloxanes, silicon polyether, hydrosilylation, microbiological colonization, building material protection

## Abstract

In the presented study, the effectiveness of a siloxane polyether (HOL7) coating on glass against microbiological colonization was assessed using microalgae as a key component of widespread aerial biofilms. The siloxane polyether was successfully synthesized by a hydrosilylation reaction in the presence of Karstedt’s catalyst. The product structure was confirmed by NMR spectroscopy and GPC analysis. In addition, the thermal stability of HOL7 was studied by thermogravimetric measurement. Subsequently, the surfaces of glass plates were modified with the obtained organosilicon derivative. In the next step, a microalgal experiment was conducted. A mixture of four strains of algal taxa isolated from building materials was used for the experiment—Chlorodium saccharophilum PNK010, Klebsormidium flaccidum PNK013, Pseudostichococcus monallantoides PNK037, and Trebouxia aggregata PNK080. The choice of these algae followed from their wide occurrence in terrestrial environments. Application of an organofunctional siloxane compound on the glass reduced, more or less effectively, the photosynthetic activity of algal cells, depending on the concentration of the compound. Since the structure of the compound was not based on biocide-active agents, its effectiveness was associated with a reduction in water content in the cells.

## 1. Introduction

Thanks to their unique structure, silicon polyethers are undoubtedly one of the most important groups of modified polysiloxanes [1]. They are composed of a siloxane chain, which, in addition to hydrophobic methyl groups, also contains hydrophilic polyether groups [2,3]. While retaining the characteristics of siloxanes, silicone polyethers are also highly hydrophilic [4,5,6]. By combining the specific properties of both components, i.e., siloxane (showing high chemical and oxidation resistance, and stability when exposed to atmospheric elements) and hydrophilic polyether groups, it is possible to obtain materials with a wide range of applications [7,8,9,10,11,12]. Therefore, they are commonly used as surfactants, e.g., in the production of polyurethane foams. Additionally, silicone polyethers are used as emulsifiers in cosmetic and pharmaceutical formulations [13,14]. They are also used as superwetting agents. Due to their behavior and appropriate orientation at the phase boundary, they strongly influence the wetting process of hydrophobic polymers, e.g., polyethylene [4,15,16]. Silicone polyether surfactants also have a bacterial effect [17,18,19]. Therefore, they are used, for example, to obtain bacterial cellulose [20] or antifouling agent [21,22]. Taking into account the above facts, how organofunctional siloxane affects the biocorrosion process is of interest. In the aeroterrestrial environment, surfaces of building materials are exposed to microbiological colonization. Bacteria, fungi, and microalgae can easily be spread by wind from one spot to another. Among these groups of microorganisms, the most persistent are aerophytic algae due to their photoautotrophic nature and ability to colonize harsh microhabitats, transforming them into more suitable or heterotrophic organisms [23]. One of the main factors increasing the success of algal colonization is the porosity of a building material [24]. However, algae can survive and develop on glass, creating green biofilms firmly attached to its smooth surface [25,26]. The planar structure of biofilm is a very complex system that includes coccoid and filamentous forms with their native bacterial microflora. The cell adhesion and biofilm development on glass surfaces are possible due to the expression of some genes associated with the attachment to abiotic, non-nutritive surfaces such as glass by regulating the secretion of extracellular polymeric substances (EPSs) comprising proteins, polysaccharides, and lipids [27].

Algal biofilms are involved in the biodeterioration of building materials [23,24,28]. Their growth affects the structure and chemical composition of wood, plaster, and brick, lowering their durability. Although there are no reports on the effect of algal growth on the technical state of glass surfaces in the land environment, their participation in aesthetic deterioration is undoubted. There are many tools used in conservation practices to protect surfaces against algal colonization. Many of them mention hydrophobic compounds like ‘silicones’, for example, alkyl silanes or alkyl alkoxy siloxanes, to be effective in preventing the adhesion and formation of biofilm [29]. As algae are poikilohydric organisms, their metabolism highly depends on the amount of water in the surrounding environment, i.e., in the form of water vapor [25]. In general, hydrophilic compound coatings attract water, making them more attractive to microorganisms. In the case of microalgae, such compounds should not have any positive effect on limiting the development of biofilms due to increased substrate humidity. 

This work presents a completely different direction of applications of silicon polyethers. In the presented study, the effectiveness of a silicon polyether (HOL7) coating on glass against microbiological colonization was assessed using microalgae as a key component of widespread aerial biofilms. In this experiment, we wanted to check how a compound with a short substituent with one hydrophilic group affects the biological activity of photosynthetic microorganisms growing on glass substrates. Hydrophilic compounds covering building materials can, on the one hand, increase the humidity of the substrate, which is not important in the case of glass substrates, and, on the other hand, absorb water suspended in the air. Photosynthetic cells absorb and periodically store water in air with higher humidity, which is why the biofilm covering the substrate may be characterized by higher humidity. Therefore, we try to answer whether there is a chance that the hydrophilic layer can absorb water from the cells, leading to a decrease in the metabolic activity of photosynthetic organisms. It is proven that silicone polyethers can be used as effective protective coatings to prevent biological corrosion of glass.

## 2. Results

### 2.1. Results of Synthesis

**Scheme 1:**
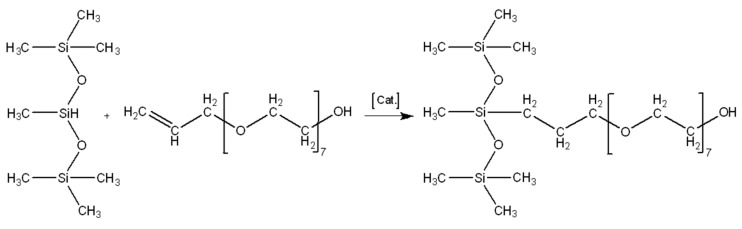
Hydrosilylation of allyl ether with 1,1,1,3,5,5,5-heptamethyltrisiloxane.

The synthesis of siloxane polyether was performed using allyl polyether containing seven ethoxy groups with the terminal hydroxy group. In this way, a compound with amphiphilic properties was obtained. In addition to hydrophobic methyl groups, hydrophilic polyether groups were attached to silicon. All reactions were carried out in toluene as a solvent. The catalyst was the commercially available Karstedt one. The hydrosilylation reaction is shown in Scheme 1.

The isolated product was subjected to spectroscopic analysis (^1^H-NMR, ^13^C-NMR, ^29^Si-NMR); the results are presented below (S1):

**^1^H NMR** (CDCl_3_, TMS) δ (ppm): -0.13 (-Si(CH_3_); -0.06 (-Si(CH_3_)_3_); 0.32 (-SiCH_2_-); 1.46 (-CH_2_CH_2_CH_2_-); 3.27 (-CH_2_O-); 3.45–3.51 (-OCH_2_CH_2_-); 3.55 (-OH). 

**^13^C NMR** (CDCl_3,_ TMS) δ (ppm): -0.75 (-SiCH_3_); 1.48 (-Si(CH_3_)_3_); 13.10 (-SiCH_2_-); 22.79 (-CH_2_CH_2_CH_2_-); 61.17–69.61 (-CH_2_O-); 70.18 (-OCH_2_CH_2_-). 

**^29^Si NMR** (CDCl_3,_ TMS) δ (ppm): -21.74 (-Si(CH_3_)CH_2_); 7.00 (-Si(CH_3_)_3_).

**Figure 1:**
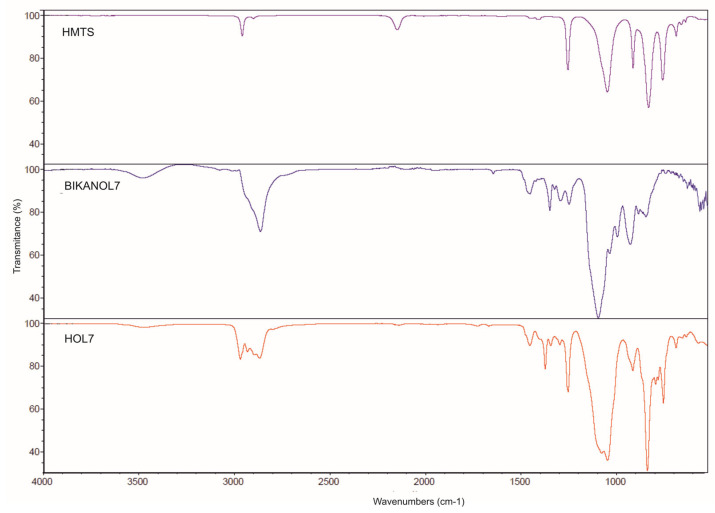
FT-IR spectra of the parent compounds and HOL7 (HMTS—1,1,1,3,5,5,5-heptamethyltrisiloxane; BIKANOL7—allyl polyether; HOL7—product).

In the FT-IR spectrum of the HOL7 product, which is shown in Figure 1, a band visible at ύ = 3500 cm^−1^ can be ascribed to stretching vibrations of the hydroxyl group. The presence of this band in the product spectrum and the decay of the bands at ύ = 2100 and 903 cm^−1^, originating from stretching vibrations of the Si-H group (present in the parent compound spectrum), testify to the formation of the hydrosilylation product and not to the occurrence of a condensation reaction between the Si-H and -OH groups. The structure of the product was also confirmed by the presence of the bands attributed to symmetric and asymmetric stretching vibrations characteristic of C-H bonds of methyl and methylene groups in the region ύ = 2700–3000 cm^−1^, as well as the bands assigned to stretching vibrations of C-O-C bonds present in the polyether chains and asymmetric stretching vibrations of Si-O-Si groups in the region ύ = 1000–1200 cm^−1^.

The molar mass distribution of the product was determined by GPC. The average molecular weight (MV) of functionalized siloxane was 974 g/mol, with a polymer dispersity index (PDI) of 1.27, indicating that the molecular weight distribution of the product was relatively uniform, (S2).

The thermogravimetry of the product was also investigated. In the temperature range up to 200 °C, the first weight loss was observed due to the evaporation of solvents and other volatile fractions, which indicated the probable beginning of sample decomposition or evaporation (by 12.30%). The next weight loss was indicative of the thermal decomposition of the sample (by 88.00%) up to approximately 500°C. In the temperature range of 500–800 °C, the weight loss was minimal (0.1446%), (S3).

Differential scanning calorimetry (DSC) analysis of the synthesized compound was also performed. In the first cycle of heating and cooling of the sample, an endothermic melting signal (−11.28 °C) and an exothermic solidification signal (−26.94 °C) were visible. There were no additional signals indicating other thermal processes taking place in the sample. In the second cycle of the thermogram, we also observed only the endothermic signal from melting (−9.78 °C) and the exothermic signal from crystallization (−26.91 °C), (S4).

### 2.2. The Effect of 5% and 10% HOL7 on Algal Biofilm

**Figure 2:**
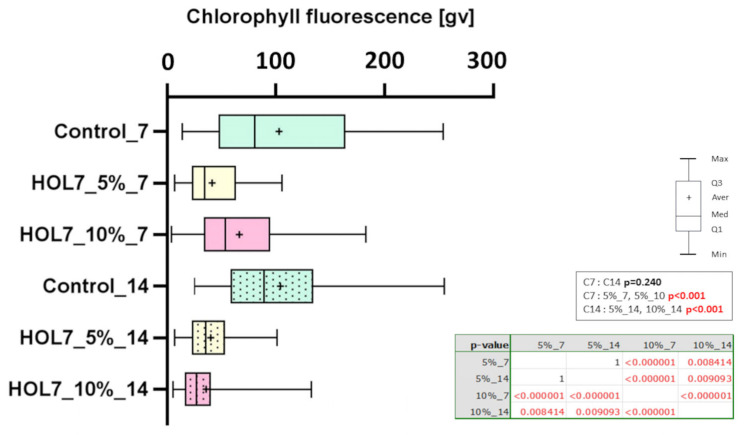
Changes in ^chl^FI of microalgal cells growing on uncoated glass substrates (control) and on those coated with 5% and 10% of HOL7 preparations (tested samples) for 7 and 14 days of cultivation.

The microalgal cells growing on control glass substrates did not reveal any significant change in fluorescence intensity of the photosynthetically active chlorophyll pigment (^chl^FI) during the 14 days of cultivation (*p* = 0.260), while all biofilms reacted to the HOL7 coatings in both concentrations with a decrease in ^chl^FI (Figure 2, Table S1). After 7 days, the preparation with HOL7 at 5% concentration suppressed the ^chl^FI by more than 10%; however, with time, there was no significant change in the effect of 5% HOL7 (*p* = 1). In most of the cells (Q3) growing for 14 days on glass with 10% HOL7, the ^chl^FI decreased up to 69.7% relative to control cells; however, there were some cells with higher ^chl^FI than in the biofilm growing on the glass with 5% HOL7. The latter compound suppressed the cells’ maximum ^chl^FI by 60.3% relative to the control, while the 10% HOL7 coatings only did so by 48.1%

**Figure 3:**
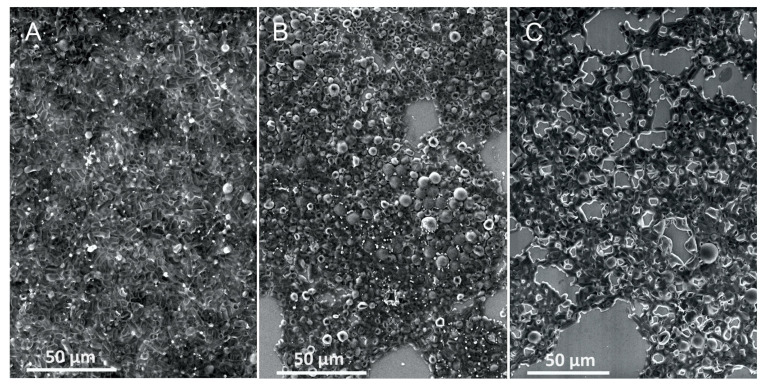
SEM photomicrographs of microalgal biofilms growing for 14 days on glass: (**A**) control, (**B**) 5% HOL7 coating, (**C**) 10% HOL7 coating. Scale bar—50 µm.

SEM photomicrographs of the biofilms after 14 days of cultivation on the glass with HOL7 coatings showed different stages of cells dehydration, depending on the concentration of the active compound (Figure 3). More cells growing on the glass with 5% HOL7 maintained a natural physiological shape, while those on that with 10% of the compound shrank and collapsed.

Since the coating with HOL7 at 5% concentration already suppressed ^chl^FI in a significant way after 7 days of cultivation, and the maximum ^chl^FI was maintained at the lowest level after 14 days of the experiment, further investigation was performed using HOL7 at 5% and lower concentrations.

### 2.3. The Effect HOL7 Solutions’ Concentrations and Methods of Application on Glass on Algal Biofilm

**Figure 4:**
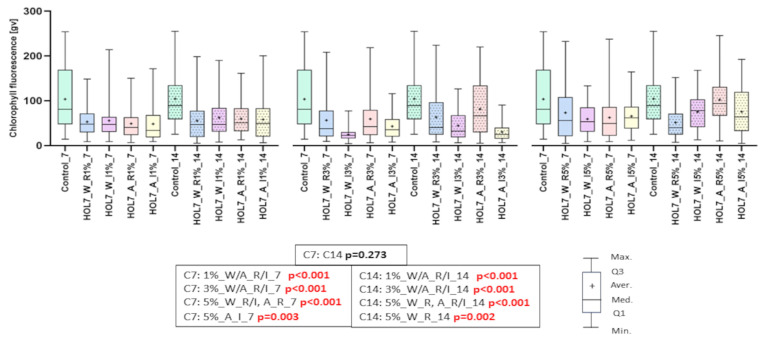
Changes in ^chl^FI of microalgal cells growing on an uncoated glass surface (control) and those coated with HOL7 in three concentrations (1%, 3%, and 5%) (tested samples) suspended in water (W) and alcohol (A) and applied on the glass substrates by immersion (I) or rubbing in (R) after 7 and 14 days of cultivation.

**Figure 5:**
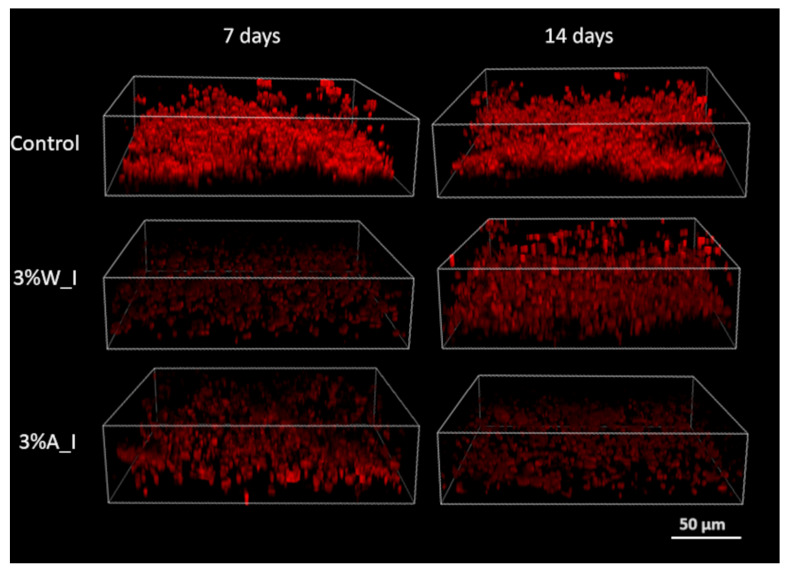
Three-dimensional CLSM visualization of algal cells in biofilms growing on the uncoated glass substrate (control) and the substrates coated with 3% HOL7 solution in water (W) or alcohol (A) by immersion (I) after 7 and 14 days of cultivation. Scale bar—50 µm.

In the second part of the experiment, the photosynthetic activity of control biofilms growing on a glass substrate did not vary significantly with time (*p* = 0.273); however, all the tested biofilm samples coated with the HOL7 solution in different concentrations revealed statistically significant changes in ^chl^FI relative to the control. However, some biofilm samples after 14 days of cultivation revealed a high level of ^chl^FI—above 85% of the control sample emission intensity (Figure 4, Table S2). In these samples, HOL7 was applied in concentrations of 3% in a water or alcohol solution or in a concentration of 5% in an alcohol solution by rubbing on the glass substrate. The average values of maximum and median ^chl^FI for the samples coated with HOL7 in different concentrations were the lowest for a 3% HOL7 solution—(^av^max/^av^med gv) *1%_7*: 170.8/42.1; *1%_14*: 187.5/48.5; *3%_7*: 155.0/34.6; *3%_14*: 165.1/40.9; *5%_7*: 191.9/55.0; *5%_14*: 189.3/68.8. After 7 days of cultivation, the highest change in ^chl^FI was detected in the biofilm growing on the glass coated by immersion in 3% HOL7 water solution (max. 77.3 gv; Q3 29.8 gv); however, with time, the photosynthetic activity in algae cells increased (max. 126.1 gv; Q3 67.8 gv). For the same HOL7 concentration and method of application, but in different solvent, the best results for all samples were obtained at the end of the experiment (7 days: max. 171.2 gv; Q3 59.1; 14 days: max 90.5; Q3: 38.9 gv) (Figure 4 and Figure 5).

**Figure 6:**
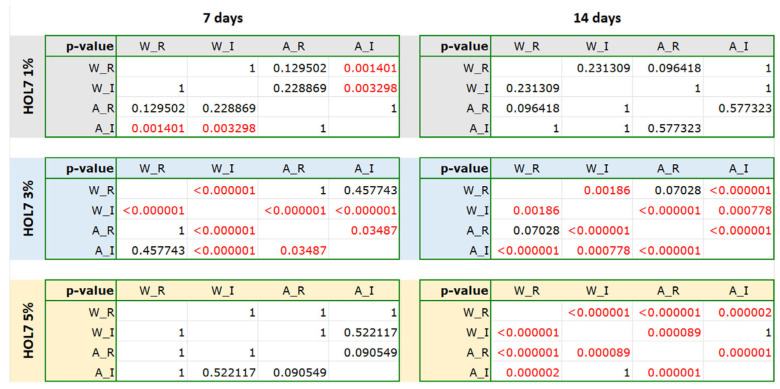
Statistical significance of the changes in ^chl^FI in algal cells growing on glass substrates coated with HOL7 in different concentrations for different solvents and methods of application; ANOVA K-W with post-hoc Bonferroni correction, *p*-level 0.05.

The effectiveness of 1% HOL7 solution in ^chl^FI inhibition after 14 days of cultivation did not differ statistically for HOL7 coatings made with different types of solvent and for different methods of application (Figure 6); however, HOL7 at this concentration did not effectively suppress the photosynthetic activity of the algal cells. Both the type of solution and method of application seem to be more important when using HOL7 at a higher concentration. In the case of rubbing the 3% HOL7 solution on the glass, the results were not significant if the water or alcohol was used as a solvent, but when using a 5% HOL7 solution, an insignificant relationship was detected in the case of immersing the glass in the solution. Nevertheless, the best result in inhibiting the photosynthetic activity of algal biofilm was achieved using a 3% HOL7 alcohol solution applied to glass by immersion.

## 3. Discussion

Various hydrophilic compounds were investigated for their efficacy against biological fouling. Mostly, the targeted bio-factors were pathogenic bacteria and fungi [30]. Application of coatings made with the use of such compounds applies a protective effect via a reduction in adhesion abilities, especially in the early stage of biofilm formation. In the experiment described, we used a mixed culture of well-formed biofilms that already had an EPS matrix and contained a much higher number of cells than the ones spread by the wind ex situ. Such inoculation is more resistant to the stress factors, and if any positive effect occurs in response to the exposure to HOL7, it will most probably be much more intense in the case of aeroterrestrial colonization.

In the aeroterrestrial environment, the existence of photosynthetic poikilohydric organisms, i.e., algae, is highly dependent on the humidity of the surrounding atmosphere. Gradual dehydration, and eventually desiccation, suppresses photosynthesis in algae, leading to photoinhibition, which is mainly caused by damage to the D1 protein subunit of the PSII reaction center encoded by psbA gene in the chloroplast genome [31]. Chlorophyll fluorescence originates close to the sites at which light energy is transformed into chemically fixed energy, and it may be significantly reduced upon the malfunction of PSII [32]. These features make ^chl^FI a unique indicator of photosynthetic performance and photosynthetic activity of cells [33,34].

Application of a bifunctional organosilicon compound on the glass reduced, more or less effectively, the photosynthetic activity of algal cells, depending on the concentration of the compound. Since the structure of the compound was not based on biocide-active agents, its effectiveness was associated with reduction of water content in the cells. Not only do the changes in ^chl^FI prove the effect, but the SEM photomicrographs also visualized a shrinkage process in the biofilms growing on the HOL7-coated glass. Reductions in cell size occur in a desiccation state, and it may reach up to 60% of the original diameter [35]. However, in the 10% HOL7-tested sample, where cells were mostly collapsed, the maximum ^chl^FI was higher than in that coated with 5% HOL7. The main goal of aeroterrestrial algal cells is to maintain and protect cellular photosynthetically based metabolism against any dehydration-concomitant damages. Their desiccation tolerance is based on a complex of mechanisms involving, inter alia, protein D1 de novo synthesis; accumulation of low molecular weight carbohydrates, like sucrose and trehalose protecting thylakoid membranes during water stress; biosynthesis; and accumulation of high concentrations of osmolytes such as polyols, betaines, proline etc., that generate low water potentials in the cytoplasm without incurring metabolic damage [36]. Among the algae strains used, *Chloroidium* and *Stichococcus* phylogenetical clades are already known for the production of glycerol erythritol, ribitol, arabitol, mannitol, sorbitol, and volemitol polyols [37], which may be responsible for the species-wide occurrence in dry microhabitats. Although the cell may easily swell or shrink depending on water accessibility, all the protective mechanisms can be activated under desiccation, maintaining the fluorescence activity at a high level. This also should contribute to drawing a careful conclusion on biofilm viability under any bio-active compounds. Since the maximum ^chl^FI biofilm is maintained at a high level, there is evidence of the presence of highly resistant cells that by the next generations may form a biofilm more immune to the effect of a compound used against biofouling.

The best result in suppressing the photosynthetic activity of algal biofilm was achieved using a 3% HOL7 alcohol solution applied to glass by immersion. This moderate concentration of solution and method of application may not increase the desiccation rate as observed upon exposure to a higher percentage of HOL7, but it may highly affect cells by the loss of the repair capacity leading to the degradation of the biofilm.

## 4. Material and methods

### 4.1. Materials 

All commercially available chemicals were used as received without any further purification. 1,1,1,3,5,5,5-heptamethyltrisiloxane (HMTS) was purchased from Sigma-Aldrich. Allyl polyether (BIKANOL7) was purchased from ICSO Chemical Production, Kędzierzyn-Koźle. The hydrosilylation catalyst was a commercially available Karstedt catalyst purchased from Sigma-Aldrich. 

### 4.2. Physicochemical Characterization

Magnetic nuclear resonance spectra: ^1^H NMR, ^13^C NMR, and ^29^Si NMR were taken on a Bruker Ascend 400, at room temperature, with CDCl_3_ as a solvent. FT-IR spectra were taken on a Nicolet iS20 Mid-Infrared FT-IR Spectrometer with a Gate diamond ATR attachment. The spectra were collected in the range 500–4000 cm^−1^, with the resolution of 2 cm^−1^, always recording 32 scans of the background and the sample. The reaction progress was quantified by observation of the rate of changes in the area of the band with a maximum at 904 cm^−1^, assigned to the stretching vibrations of Si-H.

GPC chromatograms were obtained using a Water Alliance 2695GPC system equipped with a Waters 2414 RI detector and a set of three serially connected 7.8 mm × 300 mm columns (Waters Styragel HR1, HR2, and HR4). Molecular weights and polydispersity indices were calculated on the basis of a point-to-point calibration curve of polystyrene Shodex standards in the range of 1.32 × 103 Da to 3.64 × 106 Da. THF was used as an eluent at an isocratic flow rate of 0.6 mL/min.

Thermogravimetric analysis (TGA) was performed on a Q50 apparatus (TA Instruments, New Castle, PA, USA), under nitrogen flow (60 mL/min) from room temperature to 800°C, at a heating rate of 283.15 K/min (10 °C/min).

Differential scanning calorimetry (DSC) measurements were performed on a DSC1 instrument (Mettler Toledo, Columbus, OH, USA). Analyses were made under argon atmosphere blown at a rate 20 mL/min. All samples were carefully weighed and placed in aluminum crucibles. Results were analyzed using STAR® Software provided by Mettler Toledo.

Temperature regime:

(1) Isotherm at −80 °C for 5 min;
(2) Heating to 200 °C at a rate of 10 °C/min;
(3) Isotherm at 200 °C for 5 min;
(4) Cooling to −80 °C at a rate of −10 °C/min;
(5) Isotherm at −80 °C for 5 min;
(6) Heating to 200 °C at a rate of 10 °C/min;
(7) Isotherm at 200 °C for 5 min;
(8) Cooling to −80 °C at a rate of −10 °C/min;
(9) Isotherm at −80 °C for 5 min.

Scanning electron microscopy (SEM) analyses were realized using a Quanta FEG 250 (FEI) electron microscope using a beam energy of 10 keV. All samples were analyzed without prior preparation. The microscope was operated in low vacuum mode at a pressure of 70 Pa for most of the analyses, except for the wood samples. Due to the significant sample humidity, the wood analyses were realized at a slightly elevated pressure of 100 Pa. The energy-dispersive spectroscopy (EDS) analyses were realized using the EDS Octane SDD detector (EDAX). The chamber pressure and the beam energy were the same as for SEM imaging. 

### 4.3. Synthesis of 3-[3-(Hydroxy)(polyethoxyy)propyl]-1,1,1,3,5,5,5-heptamethyltrisiloxane

Siloxane containing polyether groups was synthesized in a hydrosilylation reaction of 1,1,1,3,5,5,5-heptamethyltrisiloxane (HMTS) and allyl polyether containing seven ethoxy groups with the terminal hydroxy group (BIKOANOL7). The process was carried out in the presence of Karstedt’s catalyst and using toluene as a solvent. The reaction substrates are not sensitive to moisture, so the hydrosilylation reaction could be performed in an open system, which significantly facilitated the synthesis. An appropriate amount of 1,1,1,3,5,5,5-heptamethyltrisiloxane (7.61 g; 34 mmol), and stochiometric amounts of olefin (12.39 g; 34 mmol) and toluene (20 mL) were placed in a three-necked round-bottom flask equipped with a reflux, thermometer, and magnetic stirrer. Next, the mixture was heated to 110 °C. The portion of Karstedt catalyst, corresponding to 3 × 10^−5^ mol of Pt/1 mol of Si-H bonds (10 µL), was placed in the reaction flask together with olefin, siloxane, and toluene. The course of the reaction was monitored by IR spectroscopy, by observation of disappearance of the band at 904 cm^−1^, assigned to the Si-H bond in the substrate. After completion of the process, the post-reaction mixture was cooled, and the products were isolated by distilling off the solvent and excessive olefin under reduced pressure. The pure product was obtained at high yields of 99% (19.71 g product). The products were subjected to spectroscopic analysis to verify obtainment of the assumed structure.

### 4.4. Modification on Glass Plates

All microscope plates were cleaned by sonication in a detergent (15 min), distilled water (15 min), and acetone (15 min) and dried at 50 °C for 30 min. In the first method, the substrates were dipped in 1%, 3%, 5%, or 10% solutions of HOL7 in ethanol or water solvent for 20 min and dried at 120 °C for 60 min. The undipped parts of clean substrates after drying were polished with a cotton cloth, always using the same amount of modifier. Next, all the samples were subjected to the microalgal tests. 

### 4.5. Microalgal Experiment

The microalgal activity investigation was conducted in two steps. In the first step, the effect of the HOL7 compound in two concentrations, 5% and 10%, dissolved in alcohol, and applied on glass substrates by immersion was tested. The results provided the information that the inhibitory effect was greater when using HOL7 at a concentration of 5%. Therefore, in the subsequent part of the experiment, HOL7 was used at a concentration of 5%, 1%, or 3%. The coating was prepared using HOL7 in alcohol (A) or water (W) solvent and applied on the tested glass surface by two different methods of application—immersion (I) and rubbing in (R).

**Figure 7:**
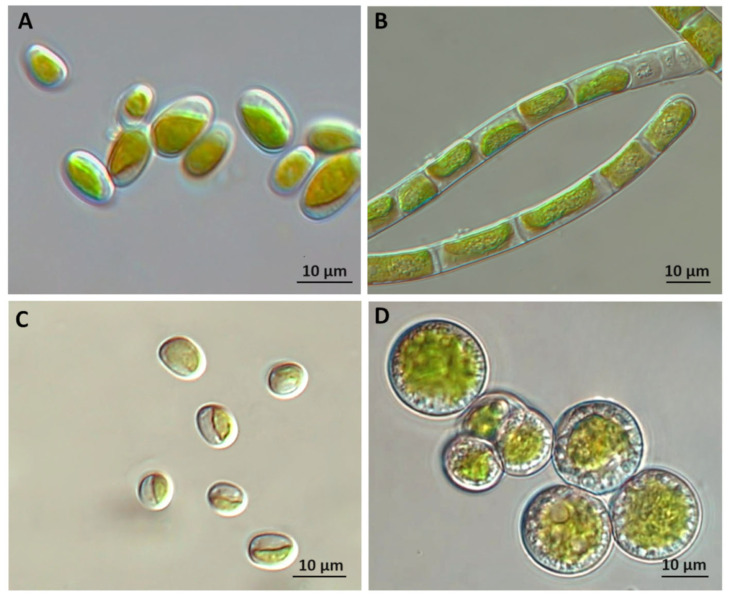
Algal strains used in the experiment: (**A**) *Chlorodium saccharophilum* PNK010; (**B**) *Klebsormidium flaccidum* PNK013; (**C**) *Pseudostichococcus monallantoides* PNK037; (**D**) *Trebouxia aggregata* PNK080. Scale bar—50 µm.

A mixture of four strains of algal taxa isolated from building materials in the quantity was used for the experiment—*Chlorodium saccharophilum* PNK010, *Klebsormidium flaccidum* PNK013, *Pseudostichococcus monallantoides* PNK037, and *Trebouxia aggregata* PNK080 (Figure 7). The choice of these algae followed from their wide occurrence in terrestrial environments; they form biofilms on different types of substrates (i.e., wood, brick, plaster, plastic, etc.) in both humid and dry microhabitats [24]. The glass surfaces were inoculated with 500 µL of the algal mixture by pipetting and cultivated for 14 days under laboratory conditions optimal for algal growth (artificial light from fluorescent tubes of 2800 Lux in a 16 h/8 h day/night period, with a temperature of 22 ± 0.2 °C and air humidity of 60 ± 5%). Photomicrographs of the strains were taken using a Nikon Eclipse 50i Light Microscope with DIC optics (Precoptic Co., Warsaw, Poland) and an Opta-Tech documentation system.

The effect of HOL7 coatings on the glass substrate was evaluated based on the changes in algal biological activity, which can be directly expressed by the photosynthetic activity of cells via chlorophyll fluorescence measurements. The analysis was based on the pigment fluorescence intensity (^chl^FI) measurements after 7 and 14 days of incubation relative to control samples—glass substrates without organosilicon compound coatings. In addition, in the first step of the experiment, SEM photomicrographs of biofilms were taken for control, 5% HOL7, and 10% HOL7 after 14 days of cultivation. The ^chl^FI measurements were made using the Leica TCS SP8 confocal microscope (Lecia Microsystems, Wetzlar, Germany) in the Laboratory of Microscopic Imaging and Specialized Biological Techniques, University of Lodz. The chlorophyll fluorescence excitation was induced using a white light laser (WLL) of 488 nm, while the detection was recorded in the PMT channel at a 620–670 nm wavelength. Each sample was recorded/scanned on the xyz axis. Measurements were performed for the depth from which the highest intensity of fluorescence emission was obtained. For each sample, 225 measurements were performed using LAS-AF 3.3.0.10134 software. To find the statistical significance of the ^chl^FI changes, the distribution of data was checked using a Shapiro–Wilk test for normality. As the data had a non-normal distribution and were independent, statistical significance of the changes between control samples after 7 and 14 days was checked using the Mann–Whitney *U* test, while for the multi-comparisons of samples, a Kurskal–Wallis one-way analysis of variance by ranks (ANOVA K-W) supported with the post-hoc Bonferroni correction was used. The statistical analyses were performed with PQStat v. 1.6.2 software, at a significance level < 0.05, while visualization of the data was made in GraphPad Prism v. 8.0.0 for Windows (GraphPad Software, San Diego, CA, USA).

## 5. Conclusions

The above-presented analyses and interpretations permit drawing the following conclusions: An effective method was developed for the preparation of functionalized polysiloxanes containing functional groups. The method is based on the hydrosilylation of olefins catalyzed by a Karstedt catalyst. The isolated product was subjected to spectroscopic analysis (^1^H NMR, ^13^CNMR, and ^29^SiNMR). A GPC measurement was also performed to analyze the size and molecular weight distribution of the product. In addition, thermal stability tests of the product were carried out. The product structure was confirmed. Next, the surfaces of glass plates were modified with the obtained organosilicon derivative that contained the hydrophilic groups enabling the formation of bonds to the substrate (polyether group). The modification was performed using two methods: (a) immersion (I) and (b) polishing with a cotton cloth (R). The substrates were dipped in 1%, 3%, 5%, or 10% solutions of HOL7 in ethanol (A) or water (W) solvent. In the next step, a microalgal activity investigation was conducted. The best result in suppressing the photosynthetic activity of algal biofilm was achieved using a 3% HOL7 alcohol solution applied to glass by immersion. This moderate concentration of solution and method of application may not increase the desiccation rate, as observed upon exposure to a higher percentage of HOL7, but it may highly affect cells by the loss of the repair capacity leading to the degradation of the biofilm. The SEM measurement of biofilms was performed after 7 and 14 days of culture on glass modified with HOL7. In summary, the synthesis of polysiloxane functionalized with a polyether group in the hydrosilylation reaction is relatively simple. Moreover, microbiological tests showed the effectiveness of the HOL7 coating against biological colonization. The obtained research results seem to be promising from the point of view of practical application.

## Data Availability

Data are contained within the article and Supplementary Materials.

## Supplementary Materials

The following supporting information can be downloaded at: https://www.mdpi.com/article/10.3390/ijms25126801/s1.
