# Supplementary figures and images for: Siloxane Containing Polyether Groups—Synthesis and Use as an Anti-Biocorrosion Coating
Source: Int J Mol Sci. 2024 Jun 20;25(12):6801. doi: 10.3390/ijms25126801 (PMC11203830; doi:10.3390/ijms25126801)

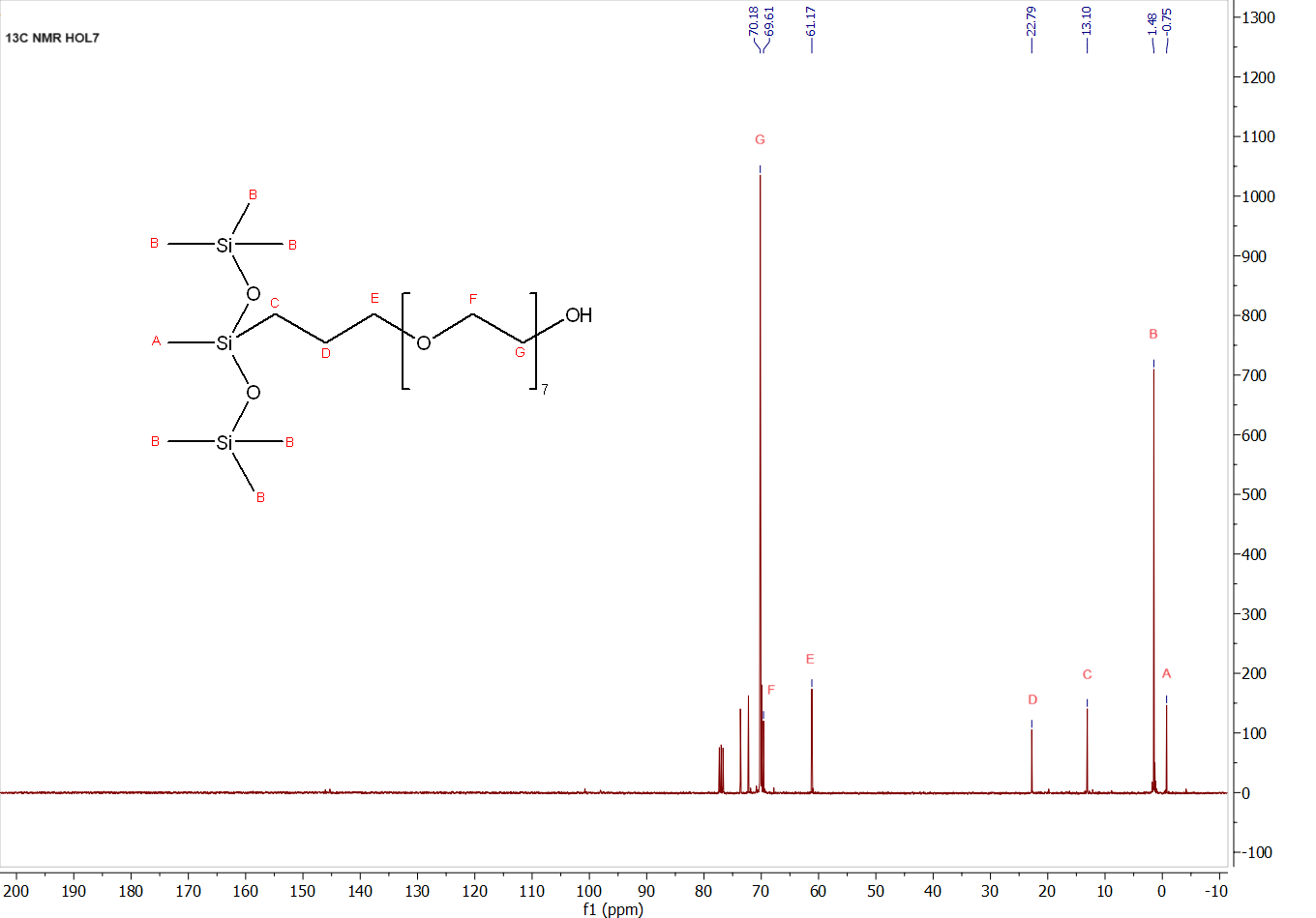

Supplement: Supplementary file 1 [file ijms-25-06801-s001.zip › Supplementary material/S1_13C NMR HOL7.tif]

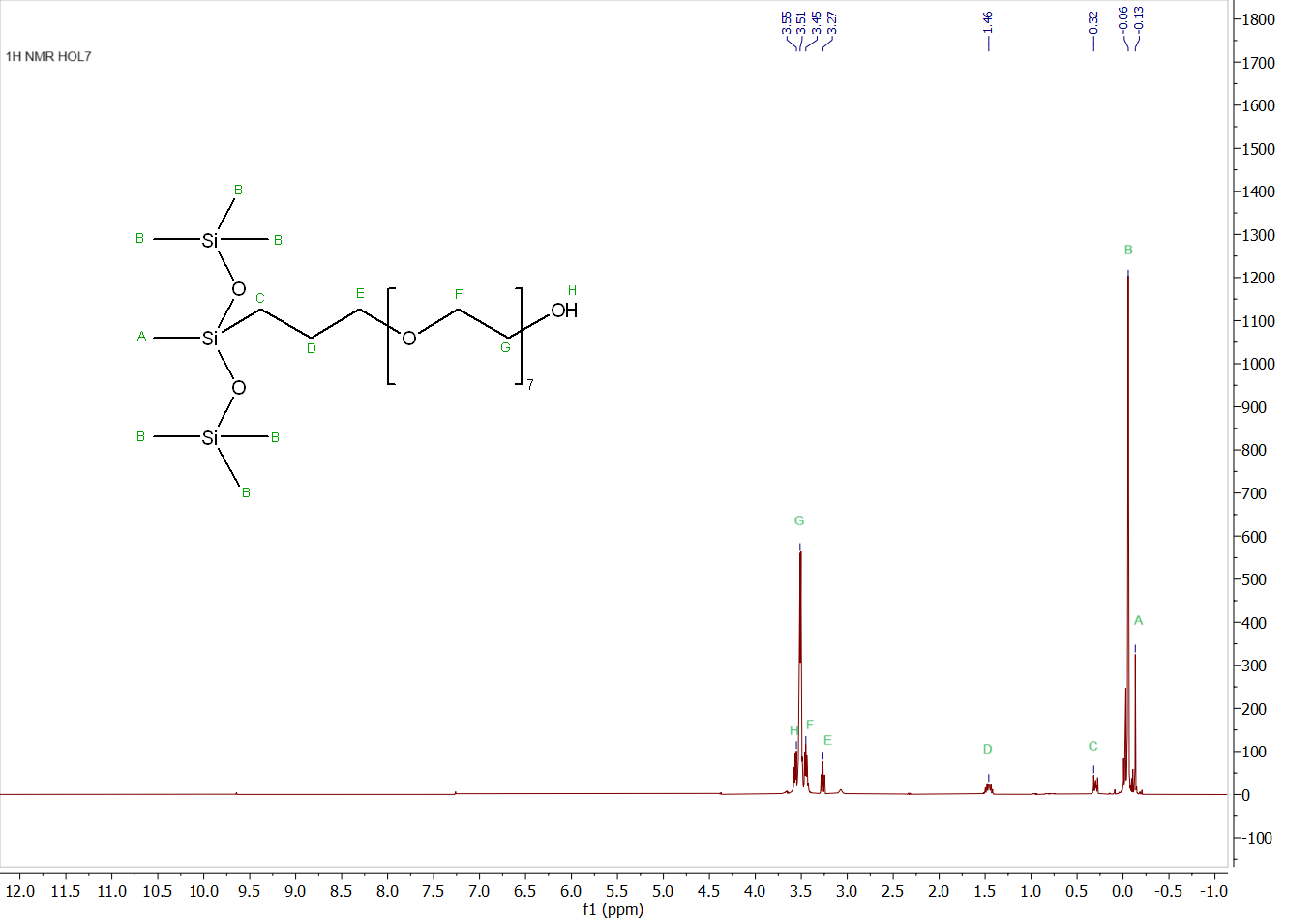

Supplement: Supplementary file 1 [file ijms-25-06801-s001.zip › Supplementary material/S1_1H NMR HOL7.tif]

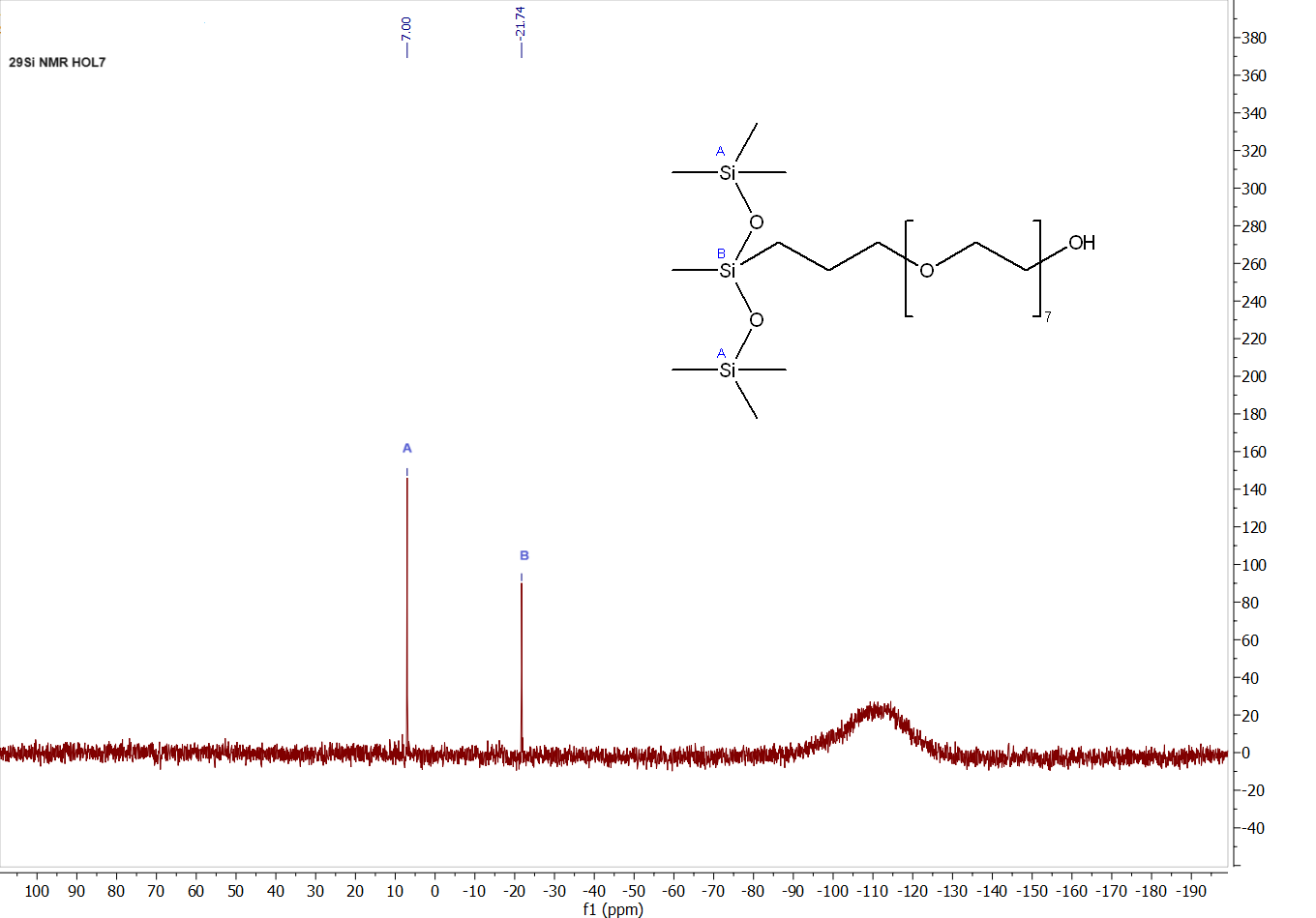

Supplement: Supplementary file 1 [file ijms-25-06801-s001.zip › Supplementary material/S1_29Si NMR HOL 7.tif]

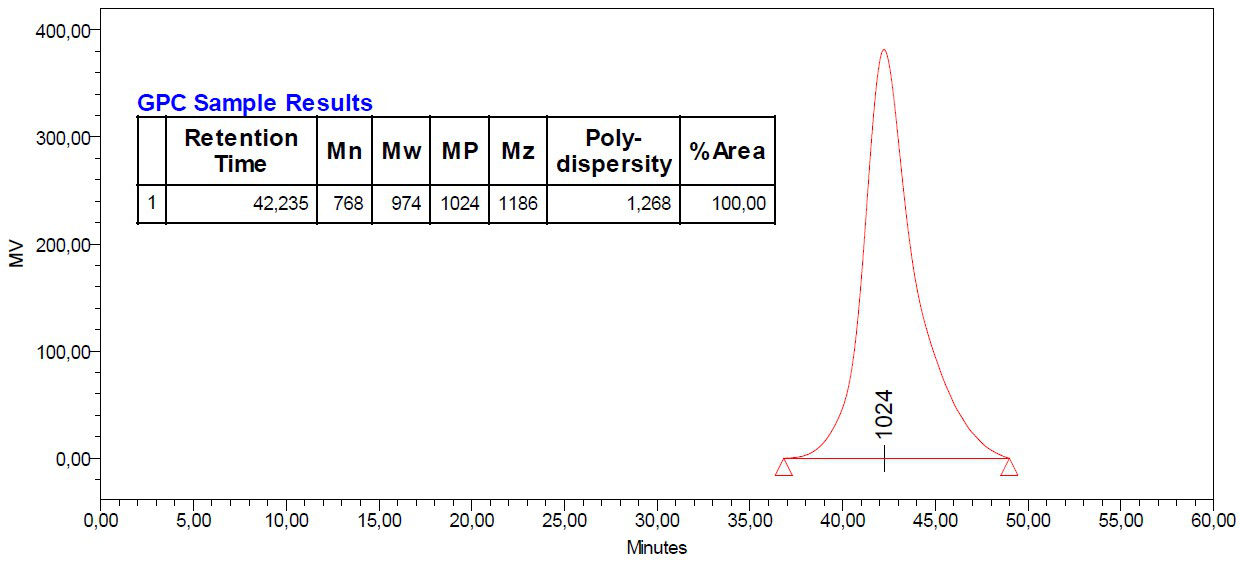

Supplement: Supplementary file 1 [file ijms-25-06801-s001.zip › Supplementary material/S2-GPC.tif]

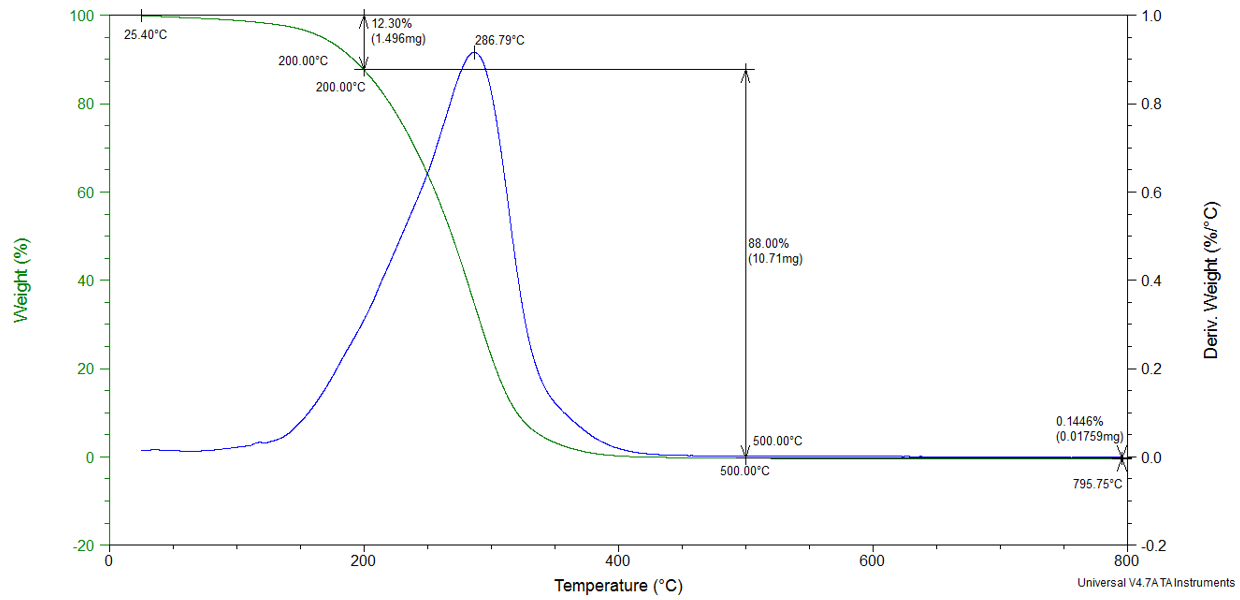

Supplement: Supplementary file 1 [file ijms-25-06801-s001.zip › Supplementary material/S3_TGA.tif]

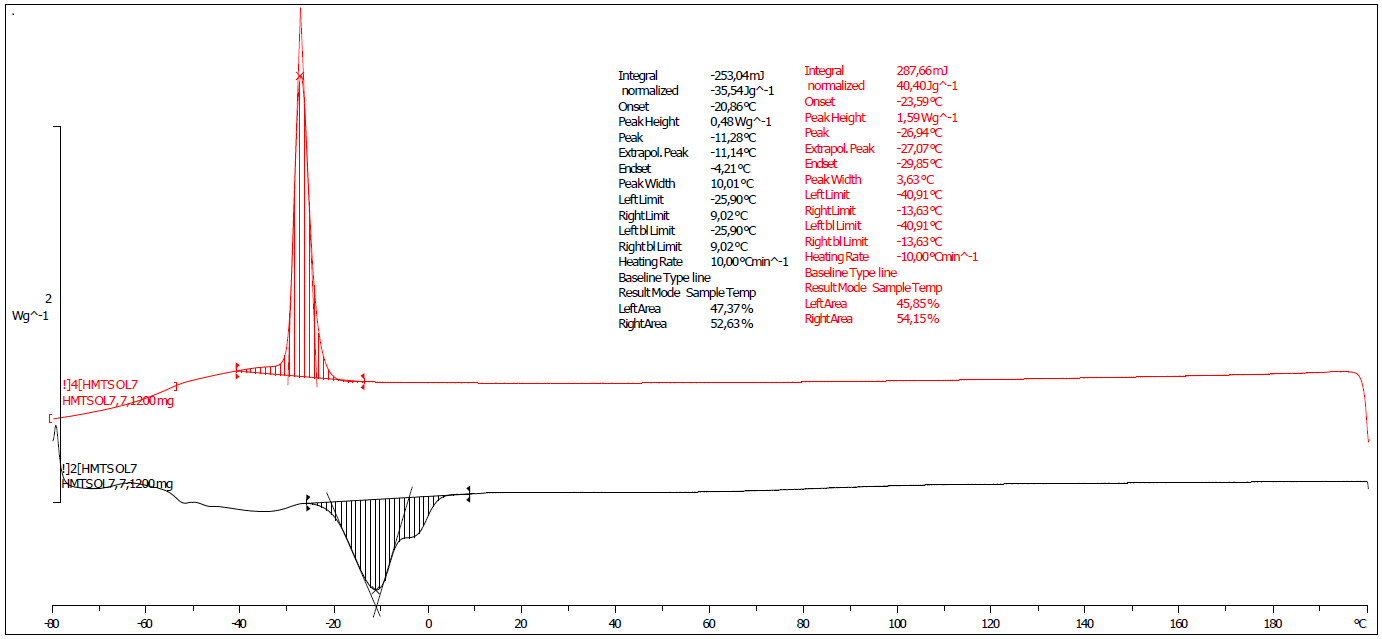

Supplement: Supplementary file 1 [file ijms-25-06801-s001.zip › Supplementary material/S4_DSC.tif]
